# Refinement of the pilocarpine-induced status epilepticus model in mice to improve mortality outcomes

**DOI:** 10.3389/fnins.2025.1592014

**Published:** 2025-05-02

**Authors:** Mi Jiang, Yu Wang

**Affiliations:** ^1^Department of Neurology, The Third Xiangya Hospital, Central South University Xiangya Medical School, Changsha, China; ^2^Department of Neurology, University of Michigan, Ann Arbor, MI, United States; ^3^Ann Arbor VA Hospital, Ann Arbor, MI, United States; ^4^Neuroscience Graduate Program, University of Michigan, Ann Arbor, MI, United States

**Keywords:** levetiracetam, pilocarpine, status epilepticus, mortality rate, mesial temporal lobe epilepsy

## Abstract

Systemic pilocarpine administration has been widely implemented to generate rodent models of mesial temporal lobe epilepsy (mTLE), but pilocarpine-induced status epilepticus (SE) in mice causes a high mortality rate, likely due to cardiorespiratory collapse associated with prolonged seizures. Although it has been well known that SE impairs the functional properties of GABARs and benzodiazepine is not effective in treating late SE both in humans and experimental animals, diazepam is still the most commonly used medication to abort SE in pilocarpine-mTLE models. Here, we instead used levetiracetam (LEV), a specific synaptic vesicle protein 2 (SV2) inhibitor in presynaptic terminals, to abort SE and achieved a substantially increased survival rate, providing a robust experimental paradigm to improve animal welfare, research cost, and experimental design. Comparable to previous studies, these mice developed reliable seizures and pathological changes in the hippocampus, including neuronal loss, gliosis, and mossy fiber sprouting. In summary, our optimized LEV-treated, pilocarpine-based protocol establishes a reliable mouse model of mTLE with significantly improved survival outcomes.

## Introduction

Mesial temporal lobe epilepsy (mTLE) is the most common type of focal drug-resistant epilepsy in adults (Spencer, 2002). Its underlying mechanisms are poorly understood, making it challenging to develop mechanistic-based treatments. Several rodent models have been developed to decode the mechanisms of its epileptogenesis, ictogenesis, and co-morbidities (Needs et al., 2019; Cronin and Dudek, 1988; Furtado Mde et al., 2002; Mello et al., 1993; Groticke et al., 2007). One of the most well-established and reliable models is to use pilocarpine intraperitoneal (i.p.) injection that induces status epilepticus (SE), leading to spontaneous seizures/chronic epilepsy after the latent period (Turski et al., 1984; Cavalheiro et al., 1991). This model is clinically valid and translatable because it recapitulates pathological features (e.g., mossy fiber sprouting, neuronal loss, dentate granule cell dispersion) (Mello et al., 1993; Mello et al., 1992), clinical features (e.g., persistent and frequent spontaneous seizures) (Baud et al., 2018), and co-morbidities (memory dysfunction and depression) (Kilpattu Ramaniharan et al., 2022; Dhaher et al., 2022). Combined with transgenic lines, the rodent pilocarpine model has become a powerful platform for epilepsy research. However, systemic pilocarpine administration in mice leads to a high mortality rate, raising ethical concerns under the 3Rs principle (reduction, replacement, and refinement) (Russell WMSaB, 1959) for humane experimental research. The high mortality rate in mice not only compromises data reliability but also presents significant challenges for experimental design. Early deaths can lead to incomplete datasets and introduce biases, especially if mortality is uneven across groups. This is particularly problematic in studies involving multiple genetic backgrounds and treatment conditions, where maintaining adequate group sizes is crucial for valid comparisons. Moreover, high mortality increases variability, reduces statistical power, and leads to inefficiencies in labor, time, and cost.

The mortality rate of human refractory SE ranges from 13% to 40%, primarily due to cardiopulmonary complications, lactic acidosis, hyperosmolarity, and multi-organ failure (Betjemann and Lowenstein, 2015; Strzelczyk et al., 2017; Neligan et al., 2019). Benzodiazepines (BZDs), such as diazepam (DZP), are established first-line drugs for the acute treatment of SE (Kienitz et al., 2022), but their efficacy decreases 20-fold in 30 min of seizures with a failure rate ranging from 10 to 55% (Migdady et al., 2022). As a family of drugs that exert their effects by allosterically modulating the activity of the ionotropic gamma-aminobutyric acid (GABA)-A receptor, BZDs increase GABA binding to the receptor, leading to Cl-channel opening and decreased neuronal excitation (Edwards and Preuss, 2025). As SE continues, time-dependent changes in receptor trafficking and internalization of synaptic GABAA receptors, along with the increase in N-methyl-D-aspartate (NMDA) receptors, could explain, at least in part, the runaway excitation, excitotoxicity, and eventually, the loss of response to BZDs (Niquet et al., 2016). In a randomized, blinded, adaptive trial to treat BZD-refractory convulsive SE, levetiracetam (LEV), a third-generation anti-seizure medication that specifically binds to synaptic vesicle protein 2A (SV2A) and inhibits calcium-dependent vesicular neurotransmitter release, displayed an equivalent efficacy to fosphenytoin or valproate with seizure cessation in approximately half the patients (Lyttle et al., 2019; Chamberlain et al., 2020). In rat pilocarpine-induced SE model, LEV injection 30 min after the onset of seizures attenuated ictal behavioral in a dose-dependent manner while pretreatment with LEV prior to pilocarpine also delayed the onset of seizures. However, the mortality rate was not documented (Zheng et al., 2010). Similarly, pretreatment with LEV in the mouse pilocarpine model increased the latencies of seizures and improved survival rate. However, the decreased mortality rate was at the expense of decreased incidence of SE (e.g., as low as 40%) (Oliveira et al., 2005).

In addition to its promising efficacy in both clinical and preclinical studies, LEV is easy to administer and has a generally favorable safety profile (Kumar et al., 2025). We hypothesize that LEV could help mitigate the high mortality rate in the mouse pilocarpine model, offering a refined protocol to improve research feasibility. In this study, we induced SE in C57BL/6 J mice using an i.p. injection of 300 mg/kg pilocarpine and terminated SE after 1 hour using 200 mg/kg LEV to assess its impact on mortality.

## Materials and methods

### Animals

6–8 weeks-old C57BL/6 J (Stock #027, Charles River Laboratories) male or female mice were housed under a 12-h light/dark cycle with food and water provided ad libitum. After pilocarpine treatment, mice were placed into cages with diet energy gel, easily accessible water, and chow provided. The cages were placed in a quiet, warm, and humid environment. All experimental procedures were conducted in accordance with the United States Public Health Service’s Policy on Humane Care and Use of Laboratory Animals and were approved by the Institutional Animal Care and Use Committee at the University of Michigan.

### Pilocarpine-induced SE model of TLE

All pilocarpine injection sessions were conducted at a consistent time each day to minimize circadian variability and ensure uniformity in experimental conditions. Prior to administering pilocarpine, each mouse received an i.p. injection of scopolamine (1 mg/kg; Sigma Aldrich) 30 min before the pilocarpine injection. This step was crucial in mitigating the peripheral cholinergic effects of pilocarpine, which can lead to unwanted side effects such as excessive salivation or gastrointestinal distress. Following scopolamine pretreatment, the mice received a single, full dose of pilocarpine (300 mg/kg; Sigma Aldrich) by i.p. injection (Mazzuferi et al., 2012) to induce seizures. The onset and progression of seizures were closely monitored and scored using the Racine scale (Racine, 1972), a widely used grading system for categorizing seizure severity in rodents. This scale is divided into five stages (Spencer, 2002; Needs et al., 2019; Cronin and Dudek, 1988; Furtado Mde et al., 2002; Mello et al., 1993), with stage 5 representing the most severe form of seizure. Stage 1 (Mild Seizures) exhibits subtle behaviors such as facial automatisms (e.g., chewing or lip-smacking) and wet-dog shakes (involuntary body movements). Stage 2 (Mild Seizures) displays more pronounced symptoms, including tail stiffening and generalized motor activity, but without overt convulsions. Stage 3 (Moderate Seizures) is characterized by low-intensity tonic–clonic seizures, typically unilateral forelimb clonus, where one forelimb shakes or spasms rhythmically. Stage 4 (Severe Seizures) involves bilateral forelimb clonus (both forelimbs shaking) accompanied by rearing, where the animal rises up on its hind limbs. Stage 5 (Most Severe Seizures), is a progression of Stage 4 seizures with the additional loss of postural tone, meaning the mouse collapses and loses coordination during the seizure, potentially leading to falls. Seizures were considered convulsive if they were Stages 3–5 on the Racine scale. SE was defined as a continuous Stage 3, 4, or 5 seizures lasting at least 5 min without subsiding for 1 h. During the experiment, all seizure behaviors and clinical features were meticulously recorded in real time to document the progression of seizures and any behavioral changes that occurred as SE developed, ensuring accurate tracking of the onset, duration, and persistence of SE and comprehensive and reproducible data.

We calculated the latency to the first convulsive seizure and the latency to SE after pilocarpine injection. We also recorded the number of pilocarpine-treated mice that (1) died acutely without SE, (2) failed to develop SE, and (3) developed SE and survived. To evaluate the efficacy of DZP (10 mg/kg) versus LEV (200 mg/kg; Sigma Aldrich, cat# L8668) treatment, mice that developed SE and survived pilocarpine administration were assigned to two treatment groups. Both treatments were administered promptly after 60 min of sustained SE, and mortality rates were recorded for each group.

### Immunohistochemistry

For immunohistochemistry, mice were placed under deep isoflurane-induced anesthesia and transcardially perfused with 0.9% phosphate buffered saline (PBS) and then with 4% paraformaldehyde (PFA) in PBS. Brains were removed and fixed in 4% PFA overnight at 4°C. Coronal sections were prepared at a thickness of 70 μm using Leica VT1000S vibratome. Sections were processed for immunohistochemistry as free-floating sections. Primary antibodies included mouse anti-NeuN (1:500; Sigma; #MAB377), mouse anti-iba1 (1:500; Abcam; #ab283319), rabbit anti-GFAP (1:500; Dako/Agilent; #Z033429-2), and rabbit anti-ZnT3 (1:250, Synaptic Systems, #197003DY5). Primary antibodies were visualized using fluorescently conjugated to Alexa Fluor 647 (Invitrogen #S21374). Nuclei were labeled with bisbenzimide (1: 500, Invitrogen, #H1398).

### Imaging and statistical analyses

Multi-channel imaging was performed using a Leica SP5 confocal microscope. Images were processed and quantification was performed using ImageJ software (NIH, United States). The image preprocessing steps included background subtraction and thresholding to improve contrast and reduce noise. Positively stained cells were detected and quantified using the “Analyze Particles” function in ImageJ, with predefined size and circularity criteria to filter out artifacts and non-cellular elements. For density quantification, the Total Area (measured in pixels^2^) was extracted to represent the cumulative stained area within the region of interest (ROI). For intensity quantification, measuring and analyzing the brightness levels of pixels within ROI to extract quantitative information. Statistical analyses were performed using GraphPad. The *p* values less than 0.05 were statistically significant. All data were shown as mean ± SEM. All images were further processed in Adobe Photoshop software.

### Electroencephalogram (EEG) implantation and analysis

EEG implantations were performed to determine whether pilocarpine-induced status epilepticus (pilo-SE) mice with LEV treatment developed spontaneous seizures. Three burr holes were drilled using a drill bit (#19007-07, 70 mm, Fine Science Tools) mounted to a surgical drill. Three epidural screw electrodes (#E363/96; P1 Technologies) were positioned and fastened through burr holes, including two electrodes were placed bilaterally on the parietal lobes, and one reference electrode was placed on the cerebellum. Electrodes were secured using dental cement (#10-000-784, Stoelting). The subdural screw electrodes were attached to a 6-pin electrode pedestal (#8K000229801F, P1 Technologies), and the entire apparatus was fixed to the skull using dental cement, as previously described (Kao et al., 2023).

Around 3 days after surgery, mice were monitored with continuous video-electroencephalogram (vEEG; Natus) for at least 7 days. Mice were placed into a square transparent plexiglass cage in a quiet room with a 12-h light/dark cycle with food and water provided. For vEEG recording, a 6-channel cable (#363-363, P1 Technologies) was connected to the 6-pin electrode pedestal and then to a commutator (#SL6C, P1 Technologies), which allowed mice to move freely in the entire cage. Video cameras pointed at cages to capture epileptic behavior in mice. Epileptiform patterns refer to brief EEG discharges that stand out from the background activity, whereas seizures are characterized by continuous epileptiform discharges that evolve rhythmically in time and space, usually persisting for more than 10 s, as previously narrated (Kao et al., 2021). Each seizure behavior was scored according to the Racine scale.

## Results

### LEV treatment improves the mortality of pilocarpine induced SE

**Figure 1:**
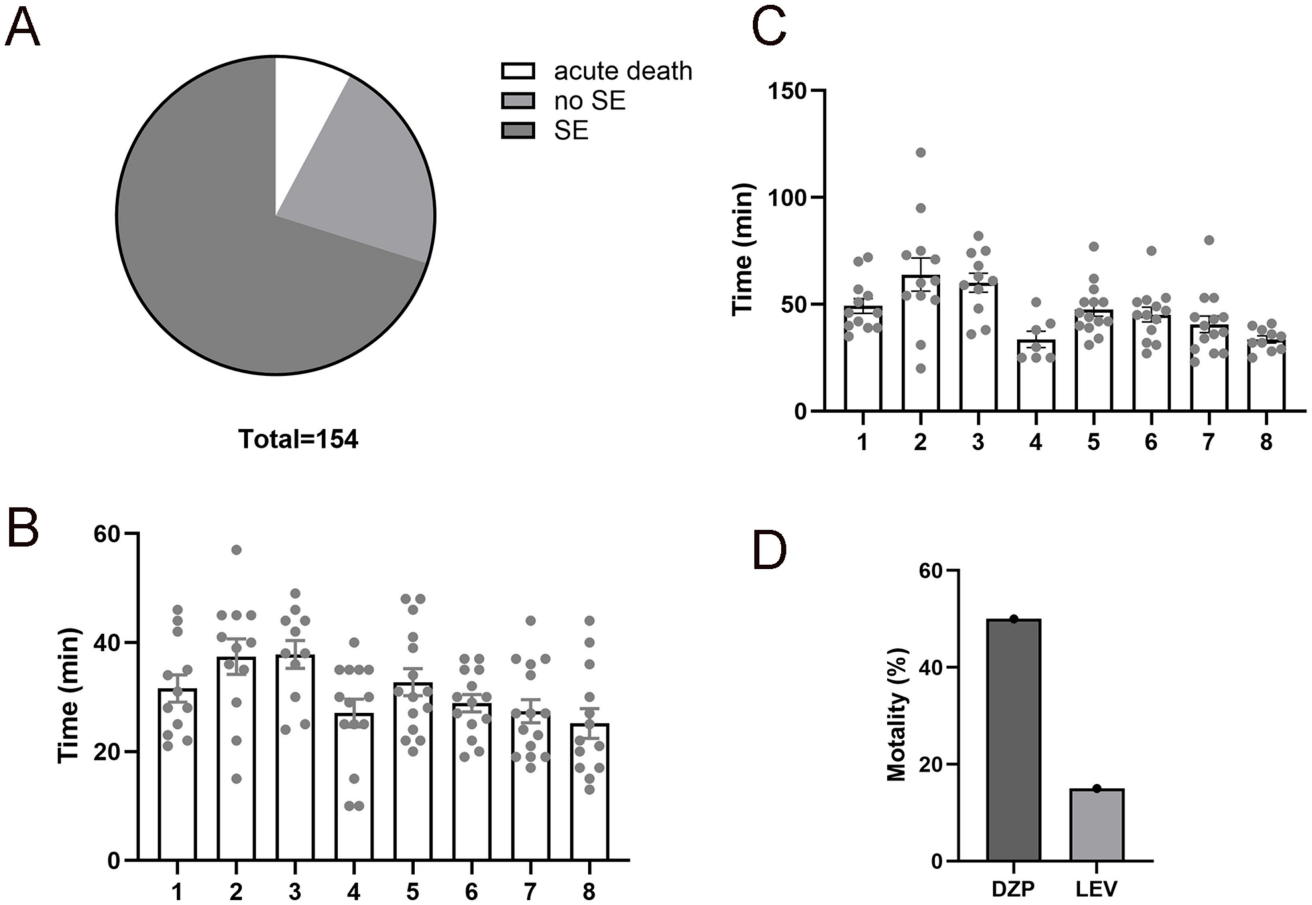
Improved mortality rate in levetiracetam (LEV)-treated pilocarpine-induced status epilepticus (pilo-SE). **(A)** The pie chart illustrates the outcomes of mice (*N* = 154) underwent pilocarpine injections, with 70.12% develop SE (dark gray), 22.07% did not develop SE (medium gray), and 7.79% experienced acute death (white). **(B)** The time interval from pilocarpine administration to the first convulsive seizure (SZ). A total of 108 mice were tested in eight different experiment sessions. Each column represents one session of pilocarpine injection, and each dot represents a mouse that received the injection. **(C)** The time interval from pilocarpine administration to the onset of SE. A total of 108 mice from the above 8 sessions were tested. **(D)** Assessment of mortality rates in mice treated with diazepam (DZP) or LEV after 1 hour of pilo-SE, showing LEV-treated pilo-SE group had a lower mortality rate (15.06%) compared to the DZP-treated pilo-SE group (50.19%). Data were shown as mean ± SEM.

To validate the efficacy of the 300 mg/kg pilocarpine dose, we assessed the SE induction rate, acute mortality, latency to the first convulsive seizure, and latency to SE onset in C57BL/6 mice. The pilocarpine injections were administered in eight sessions under the same conditions to ensure data reliability and reproducibility. Among the 154 mice that received pilocarpine injection, 108 mice (70.12%) developed full SE, showcasing the efficacy of pilocarpine in inducing SE in C57BL/6 mouse, 12 mice (7.79%) died acutely following severe generalized tonic–clonic seizures, and the remaining 34 mice (22.07%) exhibited minor, isolated, and brief seizures but did not progress to SE (Figure 1A). All 108 survived SE mice exhibited a convulsive seizure characterized by forelimb clonus and rearing (an upright posture) with a mean onset time of 30.72 ± 15.00 min post injection (Figure 1B). Mice that experienced more than three convulsive seizures progressed to SE with an average onset of 47.47 ± 17.35 min (Figure 1C).

1 hour after SE, mice were randomly assigned to receive LEV (*N* = 68) or DZP (*N* = 40) treatment to abort continuous ictal activities. LEV-treated mice exhibited a significantly lower mortality rate as compared to DZP-treated mice (15.06% vs. 50.19%, *p* < 0.01, Figure 1D), suggesting that LEV terminates prolonged SE more effectively. We also noted that LEV-treated mice had a quicker recovery from SE symptoms with improved mobility within hours post-treatment. DZP-treated mice often showed prolonged seizure activity or incomplete cessation, contributing to its higher mortality.

### Pilo-SE mice treated with LEV developed reliable and frequent seizures

**Figure 2:**
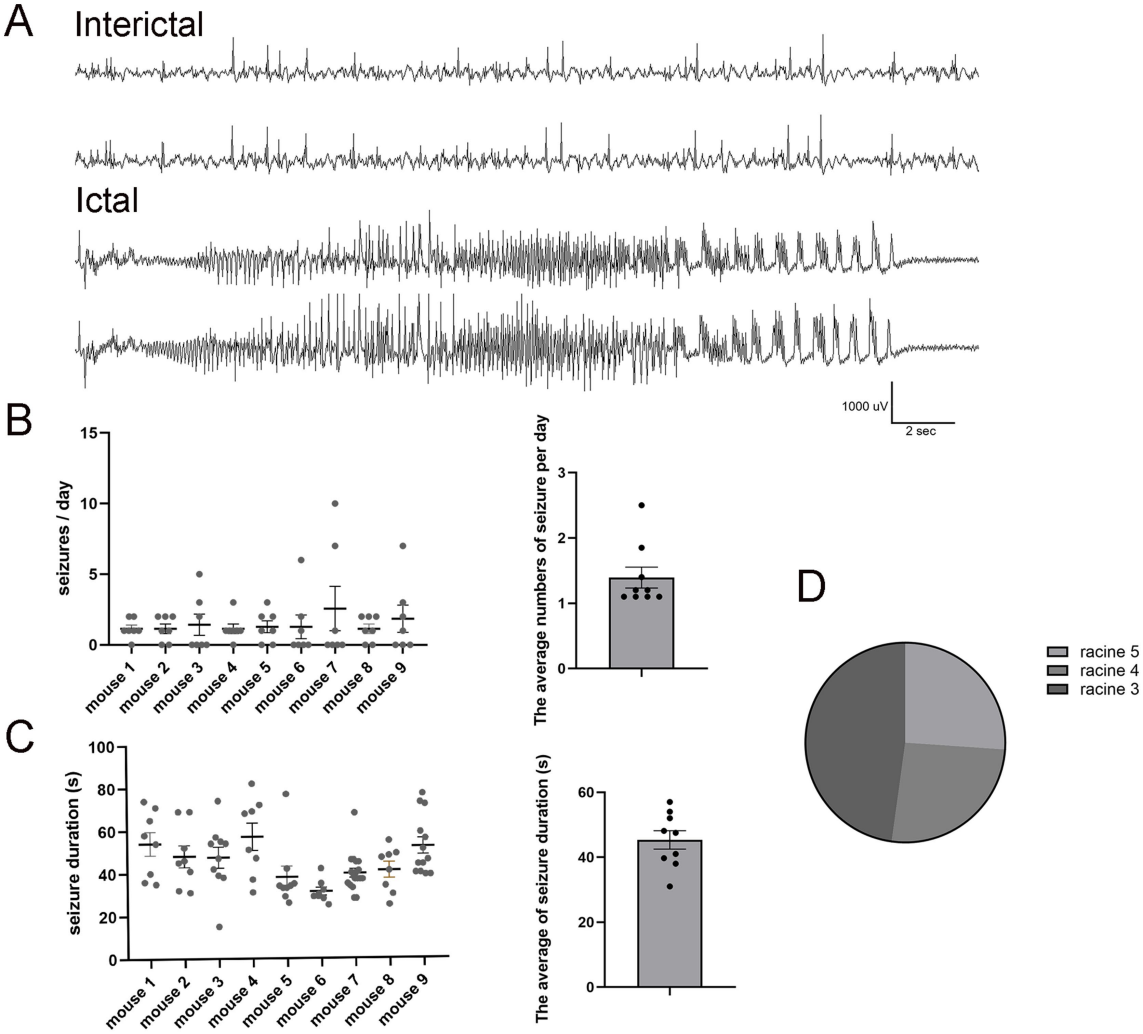
Pilo-SE mice consistently develop frequent spontaneous seizures. **(A)** Representative examples of interictal (upper panel) and ictal events (lower panel) in pilo-SE mice treated with LEV. The interictal epileptiform activities are characterized by brief, high-amplitude waveforms that occur sporadically within the background EEG activity. The ictal phase characterized by sudden onset of high-amplitude, low-voltage fast activity (beta waves), followed by a gradual increase in amplitude accompanied by rhythmic theta or delta waves over time. **(B)** The left panel illustrates seizure frequency quantified for each mouse (*N* = 9), with each dot representing a single EEG recording day over a 7-day period. The right panel presents the average number of seizures per day (1.44 ± 0.24), with each dot representing an individual mouse. **(C)** The left panel quantifies seizure duration for each mouse (*N* = 9), with each dot representing an individual seizure. The right panel shows the average seizure duration for each mouse (45.19 ± 1.56 s), with each dot representing an individual mouse. **(D)** The pie chart illustrates the percentage distribution of Racine scores for pilo-SE mice (*N* = 6). Racine Stage 3 (dark gray) account for 47.82%, while Racine Stage 4 (medium gray) and Stage 5 (light gray) each account for 26.08%. Data were shown as mean ± SEM.

To determine if the modified pilo-SE protocol still generates an mTLE mouse model, we first performed continuous vEEG recording to monitor its seizure frequency, duration, and severity. All LEV-treated pilo-SE mice (*N* = 9) developed spontaneous, recurring seizures 2–6 weeks after SE induction, consistent with previous studies (Mazzuferi et al., 2012; Peret et al., 2014). The ictal events observed in LEV-treated mice demonstrated fast frequency activities with a gradual increase in amplitude before evolving into hypersynchronous seizure onset (Figure 2A). The seizure frequency (*N* = 9, 1.44 ± 0.24 per day, Figure 2B) and the seizure duration (45.19 ± 1.56 s, Figure 2C) were comparable to those typically observed in other chronic SE models (Buckmaster et al., 2017). Among the observed seizures, 47.82% were classified as Racine Stage 3 (*N* = 6), while Racine Stage 4 and Stage 5 each accounted for 26.08% (*N* = 6) of the total seizures (Figure 2D).

### Pilo-SE mice treated with LEV developed similar pathological changes seen in human mTLE

**Figure 3:**
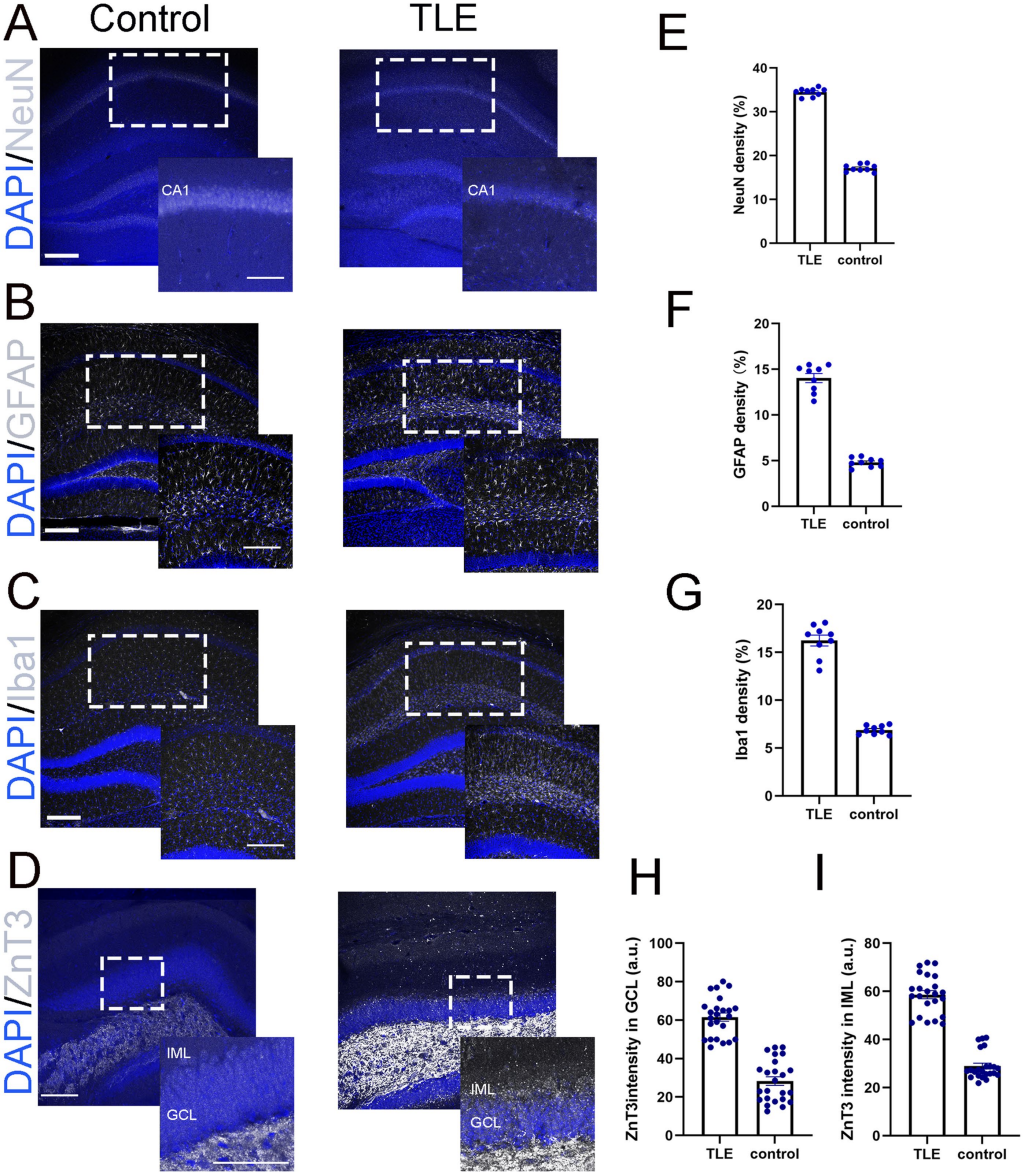
LEV-treated pilo-SE mice exhibited neuronal loss, microglial activation, astrogliosis, and mossy fiber sprouting. **(A–D)** Merged images of representative DAPI (blue, **A–D**) with NeuN (gray, **A**), GFAP (gray, **B**), Iba1 (gray, **C**), and ZnT3 (gray, **D**) showing their expression in the hippocampus under two experimental conditions: control (left panel) and temporal lobe epilepsy (TLE, right panel). **(A)** NeuN staining in the CA1 region of the control group (left panel) appears as a dense, well-organized band of neuronal nuclei, whereas the TLE group (right panel) exhibits a reduction in NeuN-positive cells, along with disrupted and fragmented neuronal layers. Scale bar: 200 μm. Insets represent a part of the CA1 (indicated with the dotted rectangle) at higher magnification. Inset scale bar 200 μm. **(B)** GFAP expression in the hippocampus is relatively low and evenly distributed in the control group (left panel), whereas in the TLE group (right panel), it is increased, with hypertrophic astrocytes exhibiting enlarged cell bodies. Scale bar: 200 μm. Insets represent a part of the hippocampus (indicated with the dotted rectangle) at higher magnification. Inset scale bar 200 μm. **(C)** Iba1 expression in the hippocampus show low and evenly distributed in the control group (left panel), whereas the TLE group (right panel) shows increased Iba1 expression with clustering of microglia. Scale bar: 200 μm. Insets represent a part of the hippocampus (indicated with the dotted rectangle) at higher magnification. Inset scale bar 200 μm. **(D)** ZnT3 expression in the hippocampus, focused on the IML and GCL layers, shows labeled mossy fibers extending from the dentate gyrus into these layers in the TLE group compare to control groups. Scale bar: 200 μm. Insets represent a part of the hippocampus (indicated with the dotted rectangle) at higher magnification. Inset scale bar 100 μm. **(E–G)** Quantification of NeuN **(E)**, GFAP **(F)**, and Iba1 **(G)** showing densities of 34.50, 14.04, and 16.24%, respectively, compared to control values of 17.13, 4.80, and 6.88%, with a significance of *p* < 0.05. **(H,I)** Quantification of ZnT3 immunofluorescence intensity revealed values of 58.64 (a.u.) in the IML **(H)** and 61.55 (a.u.) in the GCL (I), compared to control values of 28.95 (a.u.) and 28.28 (a.u.), respectively. All comparisons were made between the control and TLE groups with significant differences *p* < 0.05. IML: Inner Molecular Layer. GCL: Granule Cell Layer. a.u., Arbitrary Units. Data were shown as mean ± SEM.

We examined several key pathological features of human mTLE in LEV-treated pilo-SE mice, including neuronal loss, microglial activation, astrogliosis, and mossy fiber sprouting (Mello et al., 1992; Ammothumkandy et al., 2022). Significant neuronal loss in the hippocampus, particularly in the CA1 region, was observed (Figures 3A,E). Additionally, astrogliosis was evident in the hippocampus with increased expression of the reactive astrocytes marker, glial fibrillary acidic protein (GFAP) (Figures 3B,F). Microglial activation (Figures 3C,G), characterized by morphological changes (e.g., amoeboid shape) and increased number, was shown by immunostaining of Iba1, a microglial specific marker. In pilo-SE mice with LEV treatment, Zinc transporter 3 (ZnT3)-labeled mossy fibers aberrantly extended from the dentate gyrus into regions such as the granule cell layer (GCL) and inner molecular layer (IML) that typically do not contain mossy fibers (Figures 3D,H,I), a hallmark of hippocampal circuitry remodeling in mTLE (Sutula, 2002).

## Discussion

In this study, we developed a modified protocol for the pilocarpine-mTLE mouse model, using a single dose of LEV during the SE phase. This approach significantly improved survival rates while maintaining pathological and electroclinical features of human mTLE. Our results show that C57BL/6 mice treated with 300 mg/kg pilocarpine exhibited low acute mortality (~7%) and a high SE induction rate (~70%). The average latency to SE onset was ~47 min, consistent with prior reports of SE typically occurring within 60 min after pilocarpine administration (Vigier et al., 2021). Differences in SE response and outcomes across studies may be attributable to variations in mouse strains and protocols. For instance, NMRI mice treated with 300 mg/kg pilocarpine exhibited a 50% SE response and 90% survival rate (Mazzuferi et al., 2012). In contrast, hybrid mice receiving 350 mg/kg pilocarpine showed higher acute mortality (27.8%) and lower SE induction rates (42.2%) (Whitebirch et al., 2022).

In our study, LEV-treated mice exhibited seizure frequencies of 1.44 ± 0.24 per day, which is comparable to the previously reported average of 1.18 seizures per day in diazepam-treated mice (Buckmaster et al., 2017), suggesting that the two treatment protocols yield similar seizure frequencies in this model. Despite the lower seizure frequency in LEV-treated mice, seizures persisted into the chronic phase (6 weeks), lasting ~30–60 s. These seizures included both partial and generalized ictal semiology and exhibited electrographic features resembling those seen in TLE patients, such as fast frequency and hypersynchronous seizure onsets. Additionally, LEV-treated mice displayed key pathological hallmarks of mTLE, including hippocampal neuronal loss, reactive gliosis, and mossy fiber sprouting, reinforcing the model’s relevance for studying TLE pathophysiology.

Importantly, our protocol resulted in a low mortality rate (~15%) after administering 200 mg/kg LEV one-hour post-SE onset. Various therapeutic strategies have been proposed to reduce mortality in pilocarpine-induced SE models. For instance, NMDA receptor antagonists like ketamine have demonstrated efficacy in reducing seizure severity and mortality by mitigating excitotoxicity during SE (Borris et al., 2000). Combining ketamine with other anticonvulsants, such as propofol or midazolam, has shown potential in improving survival outcomes in lithium-pilocarpine rat models (Yilmaz et al., 2024). However, such approaches introduce additional variables and are less effective in mice, where lithium-pilocarpine combinations do not improve SE induction or survival (Muller et al., 2009). Our study demonstrated that LEV treatment effectively reduced mortality while preserving chronic TLE-associated features. This outcome aligns with LEV’s unique mechanisms, including binding to synaptic vesicle protein SV2A, modulating calcium channels (Contreras-Garcia et al., 2022), and regulating neuroprotective factors like brain-derived neurotrophic factor (BDNF) and neuropeptide Y (Husum et al., 2004). Unlike BZDs, which often fail in SE beyond 30–60 min due to GABA receptor internalization and short duration of action (Migdady et al., 2022), LEV offers a novel mechanism of action, favorable pharmacokinetics, and minimal sedative effects, making it particularly effective in benzodiazepine-resistant SE cases.

Our findings highlight that LEV significantly reduced mortality following pilocarpine injection without substantially altering seizure burden or general neuropathological outcomes. While this study primarily focused on mortality reduction, we believe our findings contribute to a growing body of research on safer treatments for status epilepticus and provide a strong foundation for future investigations into the broader effects of LEV. Our refined LEV-treated pilocarpine-SE protocol provides a reliable, low-mortality model of mTLE in mice, adherent to the “3Rs” principle by reducing animal pain and use while maintaining robust histological, electrophysiological, and behavioral characteristics of mTLE. Its simplicity, cost-effectiveness, and suitability for transgenic studies make it a valuable tool for advancing our understanding of refractory TLE and identifying therapeutic targets. However, limitations of this study include the focus on short-term outcomes, the lack of detailed characterization of seizure dynamics and behavioral comorbidities over time, and the need for further validation across different genetic backgrounds and sexes. Additionally, while LEV reduced mortality, its impact on long-term epileptogenesis and cognitive trajectories remains to be fully elucidated. Future studies should aim to address these gaps to comprehensively assess the therapeutic scope of this model.

## Data Availability

The raw data supporting the conclusions of this article will be made available by the authors, without undue reservation.
